# Non-targeted metabolomics revealed novel links between serum metabolites and primary ovarian insufficiency: a Mendelian randomization study

**DOI:** 10.3389/fendo.2024.1307944

**Published:** 2024-04-26

**Authors:** Shuang Chen, Zhaokai Zhou, Zihan Zhou, Yu Liu, Shihao Sun, Kai Huang, Qingling Yang, Yihong Guo

**Affiliations:** ^1^Center of Reproductive Medicine, The First Affiliated Hospital of Zhengzhou University, Zhengzhou, Henan, China; ^2^Department of Urology, The First Affiliated Hospital of Zhengzhou University, Zhengzhou, Henan, China; ^3^Department of Breast Surgery, the First Affiliated Hospital of Zhengzhou University, Zhengzhou, Henan, China

**Keywords:** causal effects, genetically determined metabolites, mendelian randomization, primary ovarian insufficiency, colocalization analysis

## Abstract

**Background:**

Primary ovarian insufficiency (POI) is a common clinical endocrine disorder with a high heterogeneity in both endocrine hormones and etiological phenotypes. However, the etiology of POI remains unclear. Herein, we unraveled the causality of genetically determined metabolites (GDMs) on POI through Mendelian randomization (MR) study with the overarching goal of disclosing underlying mechanisms.

**Methods:**

Genetic links with 486 metabolites were retrieved from GWAS data of 7824 European participants as exposures, while GWAS data concerning POI were utilized as the outcome. Via MR analysis, we selected inverse-variance weighted (IVW) method for primary analysis and several additional MR methods (MR-Egger, weighted median, and MR-PRESSO) for sensitivity analyses. MR-Egger intercept and Cochran’s Q statistical analysis were conducted to assess potential heterogeneity and pleiotropy. In addition, genetic variations in the key target metabolite were scrutinized further. We conducted replication, meta-analysis, and linkage disequilibrium score regression (LDSC) to reinforce our findings. The MR Steiger test and reverse MR analysis were utilized to assess the robustness of genetic directionality. Furthermore, to deeply explore causality, we performed colocalization analysis and metabolic pathway analysis.

**Results:**

Via IVW methods, our study identified 33 metabolites that might exert a causal effect on POI development. X-11437 showed a robustly significant relationship with POI in four MR analysis methods (*P*_IVW_=0.0119; *P*_weighted-median_ =0.0145; *P*_MR-Egger_ =0.0499; *P*_MR-PRESSO_ =0.0248). Among the identified metabolites, N-acetylalanine emerged as the most significant in the primary MR analysis using IVW method, reinforcing its pivotal status as a serum biomarker indicative of an elevated POI risk with the most notable P-value (*P*_IVW_=0.0007; *P*_MR-PRESSO_ =0.0022). Multiple analyses were implemented to further demonstrate the reliability and stability of our deduction of causality. Reverse MR analysis did not provide evidence for the causal effects of POI on 33 metabolites. Colocalization analysis revealed that some causal associations between metabolites and POI might be driven by shared genetic variants.

**Conclusion:**

By incorporating genomics with metabolomics, this study sought to offer a comprehensive analysis in causal impact of serum metabolome phenotypes on risks of POI with implications for underlying mechanisms, disease screening and prevention.

## 1 Introduction

Primary ovarian insufficiency (POI) is an important cause of ovarian hormone deficiency and infertility in women, and as a subclass of ovarian dysfunction, its etiology lies within the ovaries. Patients with POI suffer from irregular menstrual cycles, with abnormally high levels of gonadotropins and low levels of estrogen (1, 2). Spontaneous POI occurs in around 1% of women by age 40 and in an estimated 0.1% of women by age 30. Approximately 5% of women experience early menopause by age 45 (3). Although spontaneous ovulation occurs frequently, spontaneous pregnancy is possible in only 5% of patients, with the majority of patients with POI suffering lifelong fertility loss (4). As a consequence, POI has received worldwide attention due to its adverse effects and patients’ strong desire to fertilize, in particular among young women. In addition to causing infertility, POI is also associated with a multitude of health risks owing to chronic estrogen deficiency, including decreased bone density and increased risk of fracture, psychological impacts like anxiety, potentially early cognitive decline, etc., which poses serious implications for women’s health (1). Frustratingly, due to the highly heterogeneous and multi-aetiologic nature of POI, little progress has been made so far in characterizing the pathophysiological mechanisms underlying POI, with the biological mechanisms in 90% of cases still unclear (5). Therefore, the etiology and underlying biological processes of POI still require in-depth exploration.

Metabolites are widely distributed in human cells, tissues as well as fluids, with changes in their concentrations providing early evidence for pathological diagnosis (6, 7). Along with rapid advances in the field of metabolomics, a more systematic comprehension of an individual’s metabolic status has been achieved with genome-wide association study (GWAS) extending to metabolic phenotypes. Genetically determined metabolites (GDMs) profiles were yielded accordingly, serving as the nexus between genetic variations and environmental triggers of diseases (8–11). Notably, traditional targeted metabolomics methodology only specializes in metabolites within a confined number of pre-defined metabolic pathways; whereas the combination of GWAS with untargeted metabolomics facilitates an in-depth study to offer a novel frontier in exploitation of the disease causation (12). The emerging integration of serum metabolomics and modern genomics technologies delivers prospective insights into the genetic and metabolic mechanisms behind complex diseases (9). It is well known that multiple factors, including metabolism, might activate the pathogenesis of POI (13). Accumulated non-targeted metabolomics studies recently disclosed that impairments such as cognitive decline, anxiety, and decreased bone mineral density caused by estrogen deficiency in POI patients are causally linked with serum GDMs, strongly implicating that GDMs may shed light on the exploration of the pathogenesis (14–16). Thus, the metabolomic investigation of etiology for POI might lead to new preventive, therapeutic and management tools with great potential and clinical value.

Mendelian randomization (MR) serves as a distinctive genetic epidemiology research strategy, aiming at evaluating the causality of exposure to risk factors on disease-specific clinical outcomes (17). One of the critical basic principles in MR analysis is the utilization of instrumental variables (IVs), so that causal relationships hypothesized by studies are not solely derived from exposure factors (18). Acting as IVs, genetic variants reliably correlate with exposures and directly contribute to outcome events via the exposures (19). MR analytical methodology can provide robust and unbiased estimations of how genotypes are determined at conception (20). Upon this approach, researchers can more precisely and thoroughly probe the potential causal effects of multiple factors on disease, and target potentially vital genetic variants and metabolic pathways, laying cornerstones for future therapeutic strategies in clinical settings (21). MR analysis presents a robust alternative to address biases arising from unmeasured confounders, reverse causation, and measurement errors (22). It offers a complementary approach to randomized controlled trials (23, 24). For MR to be effectively executed, three core principles must be adhered to: firstly, genetic variants should exhibit a strong association with the exposure of interest; secondly, these variants should be independent of any confounders that could influence both the exposure and the outcome; thirdly, the genetic instruments should influence the outcome exclusively through their effect on the exposure (25, 26). Among these, the second and third assumptions, known together as the absence of horizontal pleiotropy, can be tested statistically (27). As a result, research on MR applied to GWAS data has grown in popularity over the past few decades (28, 29).

Due to the existing knowledge gap regarding the causal links between blood metabolites and POI, additional research in this field is imperative. Herein, our study employs non-targeted metabolomics to deduce causal associations between serum GDM levels and POI. Employing MR analysis on GWAS summary data, we conducted a comprehensive exploration of the causal effects of 486 blood metabolites on POI. Additionally, our research extends to colocalization and metabolic pathway analyses to uncover the underlying biological mechanisms. Our multifaceted studies not only seek to reveal the metabolic etiology associated with POI but also aim to provide profound insights into the biological processes involved, potentially paving the way for novel therapeutic interventions and prevention strategies.

## 2 Methods

### 2.1 Study design

**Figure 1:**
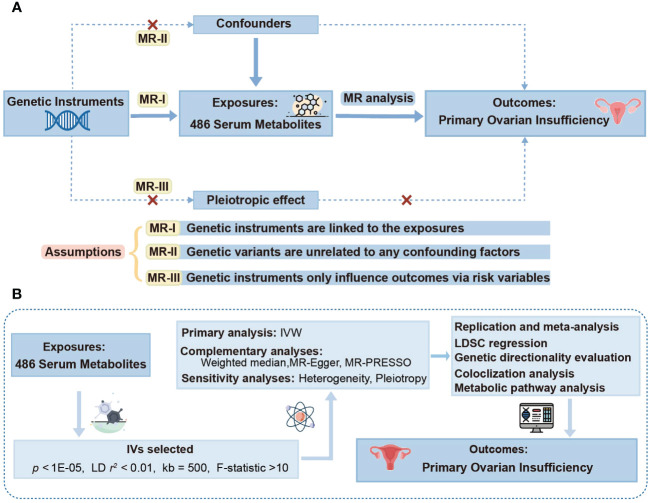
Study design overview. **(A)** Mendelian randomization (MR) analyses depend on three core assumptions. Assumption 1, genetic instruments are strongly associated with the exposures of interest; Assumption 2, genetic instruments are independent of confounding factors; Assumption 3, genetic instruments are not associated with outcome and affect outcome only via exposures. **(B)** Outline of the study design. IVW, inverse variance weighted; LD, linkage disequilibrium; MR-PRESSO, MR-Pleiotropy RESidual sum and outlier; SNPs, single nucleotide polymorphisms; LDSC, linkage disequilibrium score.

A thorough two-sample MR study was employed to probe into the causal links of 486 metabolites on POI. **Figure 1A** elucidates the study design alongside three indispensable MR assumptions: (1) genetic instruments are linked to the exposures, (2) genetic variants are unrelated to any confounding factors, and (3) genetic instruments only influence outcomes via risk variables (30). The graphical representation of our study design is depicted in **Figure 1B**.

### 2.2 Genetically determined serum metabolites

This study draws on the results of a robust analysis conducted by Shin et al. whose research offers the most comprehensive and detailed analysis of human metabolites to date, covering GWAS of 486 metabolites (11). A total of 7824 participants from two European cohorts contributed these data, and over 2.1 million single nucleotide polymorphisms (SNPs) were involved in the study. Performing a pivotal role in metabolomics GWAS data, these SNPs deepen our in-depth knowledge and mastery of the relationship between human genetic variations and serum metabolites. As shown in the Kyoto Encyclopedia of Genes and Genomes (KEGG) database, 309 of the 486 GDMs are already known and have been categorized into eight bio-chemical classes (amino acids, peptides, lipids, cofactors and vitamins, carbohydrates, energy, nucleotides, and exotic organisms), while the remaining 177 GDMs have not yet been well characterized. GWAS data for GDMs can be retrieved from Metabolomics GWAS server (https://metabolomics.helmholtz-muenchen.de/gwas/).

### 2.3 Selection of instrumental variables

To ascertain the accuracy and effectiveness of MR analyses, a rigorous selection procedure of IVs was conducted for 486 metabolites. Initially, selected SNPs significantly correlated with particular metabolites were chosen with a linkage threshold set at locus-wide significance threshold (P <1 × 10-5), which could guarantee IVs accounted for the majority of variance in metabolites. Furthermore, in order to ensure IVs independent of each other, a linkage disequilibrium threshold of r^2^ < 0.01 and a window of 500 kb were taken into account. In addition, we excluded specific palindromic SNPs so as to avoid biased results due to IVs selected inappropriately. F-statistic (β^2^exposure/SE^2^exposure) was further calculated to estimate the strength of IVs (31), and F-statistic >10 is deemed a strongly efficient selection criterion (32). Such selection criteria not only conformed to the recommendations from previous studies, but also assured the robustness and accuracy of IVs utilized in this study.

### 2.4 GWAS summary dataset for POI

GWAS summary statistics regarding POI risk were available from the FinnGen Consortium regarding 254 Finnish adult female cases and 118,228 controls, which utilized genetic data from the Finnish Biobank in combination with health records from the Finnish Health Registries. The diagnosis of POI was established according to the International Classification of Diseases-10 (ICD-10), which classifies Primary ovarian failure under code E28.3, a subcategory of Ovarian dysfunction (E28). This classification included cases with decreased estrogen levels, Premature menopause NOS (Not Otherwise Specified), and Resistant ovary syndrome, and explicitly excludes menopausal and female climacteric states (coded as N95.1), pure gonadal dysgenesis (coded as Q99.1), and Turner syndrome (coded as Q96.-). These stringent criteria ensured that the POI diagnoses used in our genetic analysis are precise and clinically validated. Given that GWAS information pertaining to POI was publicly available summary data, no additional ethical approvals were required in relation to their utilization. Data above can be publicly accessible on MRC Integrative Epidemiology Unit (https://gwas.mrcieu.ac.uk/).

### 2.5 Estimation of causal effects via MR analysis

To probe the causal association between GDMs and POI, the two-sample MR analysis was adopted in this study with a wide spectrum of methods. As the primary approach, inverse-variance weighted (IVW) method delivered consistent estimates of causal effects with all genetic variants treated as valid instrumental variables. To assure the robustness of our findings, further sensitivity tests were carried out to assess the MR estimates of latent metabolites by employing MR-Egger, weighted median, as well as MR pleiotropy residual sum and outlier (MR-PRESSO) (18).

Assessments of diversity and sensitivity were implemented to ensure consistency and reliability within our results. A variety of methodologies were applied to validate the assumption of independence, comprising MR-Egger intercept, MR-PRESSO method, etc. (33). To evaluate the presence of horizontal pleiotropy and potential bias caused by invalid IVs, MR-Egger intercept analysis was computed (34). MR-PRESSO Global test was used to provide further assessment of horizontal pleiotropy. s Q-test was employed to determine heterogeneity among SNPs, which may be attributed to horizontal pleiotropy or other biases (35). The ‘leave-one-out’ (LOO) approach demonstrated the reliability and robustness of outcomes by discarding each SNP in turn and then conducting MR analyses (34). In this way, LOO intuitively visualized whether a single SNP was driving the primary causality.

Only when the following criteria were satisfied, a strong causal relationship between GDMs and POI could be recognized in this study: 1) IVW method showed a significant difference with *P <*0.05; 2) consistent estimates were identified via the other three MR methods; 3) Cochran’s Q test, MR-Egger intercept and MR-PRESSO Global test were not significant with *P* > 0.05; 4) The MR-Steiger directionality test indicated TRUE. 5) MR estimates were not seriously disturbed by a single SNP in LOO analysis.

### 2.6 Replication and meta-analysis

To thoroughly assess the robustness of candidate metabolites identified based on the criteria outlined previously, replication analyses were conducted using alternative metabolite databases to validate the reproducibility of our findings. A comprehensive search and collection of positive metabolites were performed across databases including IEU OpenGWAS project (https://gwas.mrcieu.ac.uk/) and GWAS Catalog (https://www.ebi.ac.uk/gwas/). All collected data on matching metabolites underwent replication analysis primarily utilizing IVW method. Notably, our replication efforts also incorporated the most comprehensive GWAS study to date, which included genome-wide association analyses of 1,091 blood metabolites and 309 metabolite ratios across a cohort of 8,299 individuals (36). Subsequently, the findings from the replication analysis were integrated into a meta-analysis. A random effects model was employed in cases of significant heterogeneity (I² > 50%, H > 1.5, P < 0.05); otherwise, a fixed effects model was utilized.

### 2.7 Evaluation of genetic correlation and directionality

The accuracy of causal effect estimates in MR analysis may be compromised due to genetic associations between the exposure and the outcome of interest (37). Even after removing several SNPs associated with POI during the selection of IVs, combinations of SNPs not significantly associated with the POI may still influence its genetic predisposition to POI. To address this, we applied Linkage Disequilibrium Score (LDSC) regression, which utilizes chi-squared statistics to evaluate the genetic coinheritance of two traits, thereby assessing the genetic correlation between the identified metabolites and POI.

Moreover, to address potential biases from reverse causation, we conducted the Steiger test to more accurately determine the relationship direction between GDMs and POI (38). As a further step to validate the genetic direction, a reverse MR study was also carried out on GDMs previously identified through forward MR analysis as exerting a causal relationship with POI. This reverse analysis was performed via the same methodological framework as the forward MR, ensuring consistency in our approach.

### 2.8 Colocalization analysis

To further ascertain whether the associations of the identified GDMs between POI were driven by a locus within a genomic region, we conducted colocalization analysis via *coloc* R package. Coloc employs a Bayesian framework to generate posterior probabilities for 5 mutually exclusive hypotheses concerning the sharing of causal variants between two phenotypes. These hypotheses included H0 (neither trait has a genetic association in the region), H1 (only trait 1 has a genetic association in the region), H2 (only trait 2 has a genetic association in the region), H3 (both traits are associated, but with different causal variants), and H4 (both traits are associated and share a single causal variant) (39). H4/(H3+H4) represents the probability of colocalization conditional on the presence of a causal variant for the outcome (40).

### 2.9 Metabolic pathway analysis

To uncover the biological mechanisms through which blood metabolites influence POI causally, we conducted a detailed metabolic pathway analysis using MetaboAnalyst 6.0 (https://www.metaboanalyst.ca/). Utilizing the functional enrichment analysis module on MetaboAnalyst 6.0, we honed in on identifying key metabolite pathways, drawing from the comprehensive dataset available in the Kyoto Encyclopedia of Genes and Genomes (KEGG) database. Adhering to strict significance thresholds (P <0.05), our analysis provided a thorough and reliable examination of the metabolites associated with the POI, offering insightful revelations about its metabolic underpinnings and potential pathways of pathogenesis.

### 2.10 Statistical analysis

All MR analyses were carried out in R (4.2.0) software. Packages such as *MendelianRandomization*, *MR-PRESSO, coloc*, and *forestplot*, were employed in this study. Collectively, the array of methods and tests presented a systematic and rigorous framework to assess causal effects between GDMs and POI. If the estimated causal effect *P* value for GDMs was less than 0.05, GDMs were considered statistically significantly associated with POI and defined as latent metabolites as suggestive risk predictors for POI. A two-tailed *P <*0.05 was deemed statistically significant in all tests of sensitivity analysis. For the analysis of genetic correlation, the LDSC software was employed (41). Additionally, the online tool LocusZoom was utilized to display the results of the colocalization analysis (42).

## 3 Results

### 3.1 Strength of genetic instrumentals for 486 GDMs

With GWAS summary statistics, our study exploited two-sample MR analysis to uncover the underlying causal effects between GDMs and POI. A total of 9552 SNPs were enrolled in the study on the basis of rigorous screening criteria. SNPs for IVs corresponding to the 486 metabolites in serum ranged from 2 to 481, accounting for 0.24% to 70.82% of variances in corresponding GDMs. Of note, the minimum F-statistic for the strength of the IVs employed in this study was 17.41. The F-statistics corresponding to all SNPs were above 10, indicating that all IVs associated with the 486 GDMs were sufficient to fulfill the requirements to achieve a potent MR analysis.

### 3.2 Causality of serum metabolites on POI

**Figure 2:**
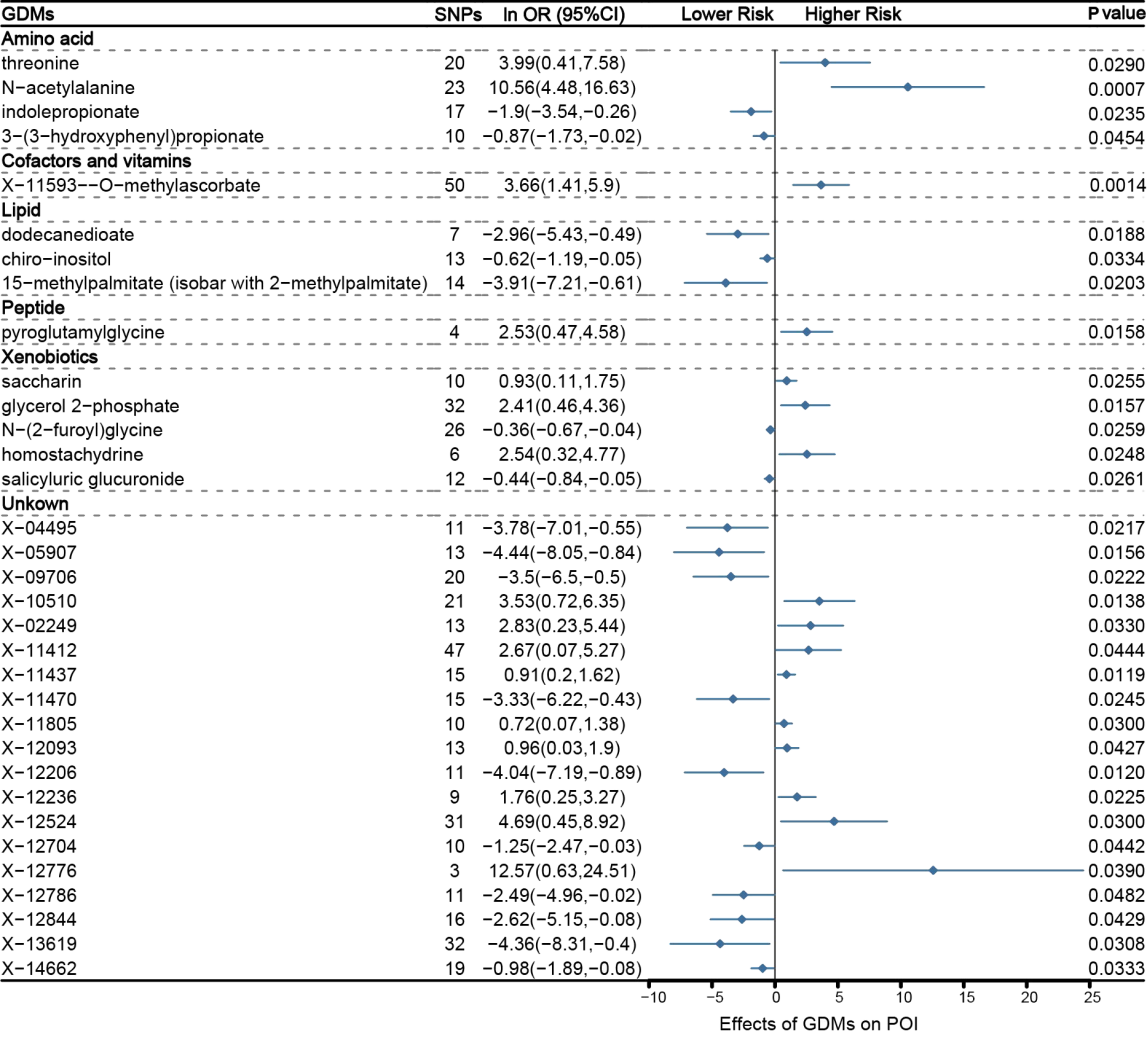
Mendelian randomization associations between serum metabolites and POI based on inverse-variance weighted (IVW) method.

Through IVW approach, 14 known GDMs and 19 unknown metabolites that significantly correlate with POI were identified (**Figure 2**). The 14 known GDMs under focused attention contained 4 amino acids, 1 cofactor and vitamin, 3 lipids, 1 peptide, and 5 xenobiotics. Notably, among these well-known metabolites, N-acetylalanine was detected to be the most prominently linked to POI (*P*_IVW_ =0.0007). With 23 SNPs serving as proxy predictors, increased levels of N-acetylalanine were strongly tied to a significantly increased risk of POI (logarithmic value of odds ratio [ln OR]: 10.56; 95% confidence interval [CI]: 4.48-16.63). Similar to N-acetylalanine, the other 15 metabolites associated with an elevated risk of POI were also determined, including threonine (ln OR: 3.99; 95% CI: 0.41-7.58; *P*_IVW_ =0.0290), X-11593–O-methylascorbate (ln OR: 3.99; 95% CI: 1.41-5.9; *P*_IVW_ =0.0014), pyroglutamylglycine (ln OR: 2.53; 95% CI: 0.47-4.58; *P*_IVW_ =0.0255), glycerol 2−phosphate (ln OR: 2.41; 95% CI: 0.46-4.36; *P*_IVW_ =0.0157), homostachydrine (ln OR: 2.54; 95% CI: 0.32-4.77; *P*_IVW_ =0.0248), etc. In contrast, 17 other metabolites were characterized to be related to a reduced risk of POI, comprising indolepropionate (ln OR: −1.9; 95% CI: −3.54- −0.26; *P*_IVW_ =0.0235), dodecanedioate (ln OR: −2.96; 95% CI: −5.43- −0.49; *P*_IVW_ =0.0188), salicyluric glucuronide (ln OR: −0.44; 95% CI: −0.84- −0.05; *P*_IVW_ =0.0261), etc. Altogether, with genetic variations as a proxy, a total of 33 GDMs with potential pathogenic effects on POI were defined on the basis of IVW method.

### 3.3 Sensitivity analysis

Even though the IVW approach is very effective for deducing causal relationships between exposure and outcome in complicated diseases, it is susceptible to the effects of weak instrumental biases. Thus, sensitivity analysis was further implemented to minimize these biases. **Table 1** shows the results of the sensitivity analysis for evaluating the stability and robustness of the metabolites identified by IVW. When sensitivity tests were employed, only 1 unknown metabolite, X-11437, was unearthed from 33 GDMs with a strong causal association with POI (*P*_weighted-median_ =0.0145; *P*_MR-Egger_ =0.0499; *P*_MR-PRESSO_ =0.0248). Besides IVW method, consistent causality estimations that serum X-11437 increased the risk of POI could also be derived from these three MR methods, involving MR-Egger, weighted median, and MR-PRESSO (**Table 1**).

**Table 1:** Sensitivity analysis of causal associations between metabolites and POI.

GDMs	MR-Egger		Weighted median		MR-PRESSO	
	lnOR (95% CI)	P-value	lnOR (95% CI)	P-value	lnOR (95% CI)	P-value
Amino acid						
threonine	8.15(-5.54,21.84)	0.2585	3.04(-1.64,7.71)	0.2150	3.99 (2.07, 5.92 )	**0.0007**
N−acetylalanine	31.44(-75.27,138.16)	0.5698	7.35(-1.08,15.77)	0.0875	10.56 ( 4.58 , 16.53 )	**0.0022**
indolepropionate	-2.03(-5.36,1.3)	0.2505	-2.04(-4.24,0.16)	0.0695	-1.9 ( -3.24 , -0.55 )	0.0140
3−(3−hydroxyphenyl)propionate	-0.84(-3.1,1.42)	0.4888	-0.73(-1.88,0.42)	0.2138	-0.87 ( -1.66 , -0.09 )	0.0574
Cofactors and vitamins						
X−11593−−O−methylascorbate	3.14(-0.92,7.2)	0.1364	2.86(-0.69,6.4)	0.1142	3.66 ( NA , NA )	**0.0010**
Lipid						
dodecanedioate	2.15(-7.17,11.47)	0.6696	-2.71(-5.89,0.48)	0.0964	-2.96 ( -4.59 , -1.33 )	**0.0120**
chiro−inositol	0.47(-0.78,1.72)	0.4735	-0.2(-1,0.59)	0.6194	-0.62 ( -1.19 , -0.05 )	0.0548
15−methylpalmitate (isobar with 2−methylpalmitate)	-5.86(-12.5,0.78)	0.1091	-3.8(-8.34,0.74)	0.1010	-3.91 ( -7.03 , -0.79 )	**0.0289**
Peptide						
pyroglutamylglycine	-1.25(-7.36,4.85)	0.7266	3.06(0.49,5.63)	**0.0198**	2.53 ( 0.47 , 4.58 )	0.0948
Xenobiotics						
saccharin	1.55(0,3.1)	0.0858	1.3(0.26,2.33)	**0.0144**	0.93 ( 0.46 , 1.4 )	**0.0038**
glycerol 2−phosphate	2.9(-0.61,6.41)	0.1162	3.15(-0.07,6.38)	0.0549	2.41 ( 0.46 , 4.36 )	0.0217
N−(2−furoyl)glycine	-0.4(-0.89,0.1)	0.1275	-0.25(-0.72,0.21)	0.2829	-0.36 ( -0.62 , -0.09 )	**0.0145**
homostachydrine	-3.75(-10.46,2.97)	0.3355	2.62(-0.48,5.71)	0.0977	2.54 ( 0.46 , 4.63 )	0.0622
salicyluric glucuronide	-0.52(-1.21,0.17)	0.1723	-0.26(-0.81,0.29)	0.3539	-0.44 ( -0.79 , -0.1 )	**0.0270**
Unknown						
X−04495	-2.46(-10.45,5.54)	0.5623	-2.25(-6.09,1.6)	0.2522	-3.78 ( -7.01 , -0.55 )	**0.0446**
X−05907	-4.53(-12.8,3.74)	0.3061	-4.58(-9.56,0.4)	0.0712	-4.44 ( -7.94 , -0.95 )	**0.0284**
X−09706	-5.1(-12.32,2.11)	0.1827	-2.38(-6.6,1.83)	0.2682	-3.5 ( -6.5 , -0.5 )	**0.0338**
X−10510	3.23(-3.17,9.64)	0.3351	4.34(0.26,8.42)	**0.0373**	3.53 ( 1.17 , 5.9 )	**0.0083**
X−02249	7.75(-0.9,16.41)	0.1067	2.89(-0.63,6.4)	0.1078	2.83 ( 0.35 , 5.32 )	**0.0448**
X−11412	5.37(-1.11,11.85)	0.1110	1.44(-2.58,5.46)	0.4833	2.67 ( 0.29 , 5.05 )	**0.0331**
X−11437	1.15(0.11,2.2)	**0.0499**	1.07(0.21,1.93)	**0.0145**	0.91 ( 0.2 , 1.62 )	**0.0248**
X−11470	-2.67(-9.76,4.43)	0.4743	-3.07(-6.63,0.5)	0.0921	-3.33 ( -6.22 , -0.43 )	**0.0411**
X−11805	0.75(-0.17,1.67)	0.1475	0.63(-0.25,1.51)	0.1612	0.72 ( 0.33 , 1.12 )	**0.0056**
X−12093	0.42(-1.27,2.11)	0.6349	0.67(-0.57,1.9)	0.2896	0.96 ( 0.19 , 1.74 )	**0.0308**
X−12206	-14.39(-25.26,-3.53)	**0.0289**	-3.63(-7.87,0.61)	0.0935	-4.04 ( -7.19 , -0.89 )	**0.0308**
X−12236	1.67(-2.63,5.96)	0.4714	1.1(-0.99,3.19)	0.3023	1.76 ( 0.25 , 3.27 )	0.0520
X−12524	2.69(-6.8,12.19)	0.5826	3.57(-2.77,9.91)	0.2697	4.69 ( 0.48 , 8.9 )	**0.0370**
X-12704	-2.22(-5.88,1.44)	0.2684	-1.62(-3.21,-0.02)	**0.0476**	-1.25 ( -2.24 , -0.26 )	**0.0355**
X-12776	6.87(-11.12,24.86)	0.5909	11.35(-2.85,25.56)	0.1171	NA	NA
X-12786	-4.2(-8.69,0.3)	0.1005	-3.06(-6.08,-0.03)	**0.0477**	-2.49 ( -4.96 , -0.02 )	0.0764
X-12844	-3.11(-12.19,5.97)	0.5129	-1.33(-4.93,2.26)	0.4676	-2.62 ( -5.07 , -0.16 )	0.0540
X-13619	-4.81(-17.08,7.47)	0.4486	-4.46(-10.37,1.45)	0.1387	-4.36 ( -7.97 , -0.74 )	**0.0246**
X-14662	-0.99(-2.54,0.56)	0.2262	-1.01(-2.3,0.27)	0.1222	-0.98 ( -1.89 , -0.08 )	**0.0474**

Values in bold represent statistically significant data with P < 0.05.

**Figure 3:**
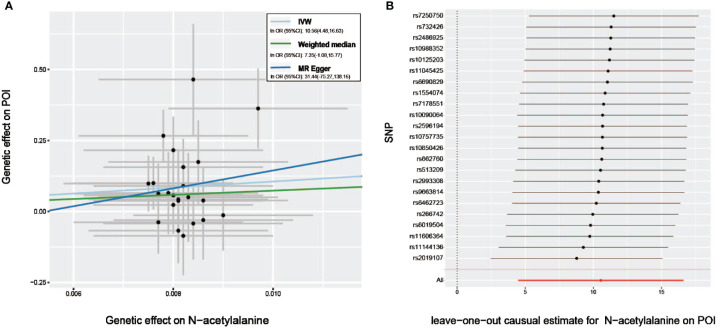
Sensitivity analysis of genetic associations of N−acetylalanine on POI. **(A)** Scatter plot of potential effects of single-nucleotide polymorphisms (SNPs) on N−acetylalanine vs. POI, with the slope of each line corresponding to the estimated MR effect per method. **(B)** Leave-one-out analysis for the impact of individual SNPs on the association between N−acetylalanine and POI risk. By leaving out exactly one SNP, it depicts how each SNP influences the overall estimate.

Furthermore, with the criteria less stringent, we inferred that all but 6 GDMs (indolepropionate, 3-(3-hydroxyphenyl) propionate, chiro -inositol, homostachydrine, X-12236, X-12776, and X-12844) of the 33 metabolites identified by IVW passed at least one additional sensitivity test (**Table 1**). Intriguingly, given that the MR-Egger and weighted median methods relied on potentially incorrect assumptions, N-acetylalanine, the most prominent known compound we identified through IVW, still demonstrated a strong correlation on the risk of POI in the MR -PRESSO test (*P*_MR-PRESSO_ =0.0022), implicating that this known GDM deserves more in-depth studies (**Table 1**, **Figure 3A**). The LOO approach demonstrated the absence of any potential outliers in IVs, which implied that no leading SNPs would severely affect the results after elimination, and further confirmed the credibility of our MR results against this critically important metabolite (**Figure 3B**).

A series of sensitivity analyses were subsequently conducted in this study to further evaluate the robustness of IVW results. For 33 serum metabolites excavated by IVW, Cochran’s Q-test showed no heterogeneity among the IVs (**Table 2**). The results of MR-Egger regression intercept and MR-PRESSO global test demonstrated that our MR analyses were not subjected to any potential effect of horizontal pleiotropy (*P >*0.05) (**Table 3**).

**Table 2:** Results of heterogeneity by the Cochran’s Q test.

GDMs	Cochran’s Q test via IVW			Cochran’s Q test via MR-Egger		
	Q	Q_df	Q_pval	Q	Q_df	Q_pval
Amino acid						
threonine	5.463	19	0.999	5.083	18	0.999
N−acetylalanine	21.257	22	0.505	21.108	21	0.452
indolepropionate	10.782	16	0.823	10.774	15	0.768
3−(3−hydroxyphenyl)propionate	7.592	9	0.576	7.591	8	0.474
Cofactors and vitamins						
X−11593−−O−methylascorbate	41.030	49	0.784	40.941	48	0.755
Lipid						
dodecanedioate	2.623	6	0.854	1.380	5	0.927
chiro−inositol	14.161	12	0.291	10.576	11	0.479
15−methylpalmitate (isobar with 2−methylpalmitate)	11.613	13	0.560	11.173	12	0.514
Peptide						
pyroglutamylglycine	3.310	3	0.346	1.668	2	0.434
Xenobiotics						
saccharin	2.996	9	0.964	2.147	8	0.976
glycerol 2−phosphate	34.143	31	0.319	34.019	30	0.280
N−(2−furoyl)glycine	17.974	25	0.843	17.925	24	0.807
homostachydrine	4.399	5	0.493	0.612	4	0.962
salicyluric glucuronide	8.382	11	0.679	8.317	10	0.598
Unknown						
X−04495	12.968	10	0.225	12.786	9	0.173
X−05907	11.301	12	0.503	11.300	11	0.418
X−09706	20.924	19	0.341	20.659	18	0.297
X−10510	14.116	20	0.825	14.106	19	0.777
X−02249	10.871	12	0.540	9.505	11	0.575
X−11412	38.511	46	0.776	37.712	45	0.771
X−11437	20.001	14	0.130	19.404	13	0.111
X−11470	20.488	14	0.115	20.425	13	0.085
X−11805	3.240	9	0.954	3.232	8	0.919
X−12093	8.241	12	0.766	7.669	11	0.743
X−12206	11.241	10	0.339	7.471	9	0.588
X−12236	8.285	8	0.406	8.282	7	0.308
X−12524	29.667	30	0.483	29.451	29	0.442
X-12704	5.968	9	0.743	5.664	8	0.685
X-12776	0.912	2	0.634	0.223	1	0.637
X-12786	15.065	10	0.130	13.836	9	0.128
X-12844	14.068	15	0.520	14.056	14	0.446
X-13619	25.914	31	0.725	25.908	30	0.680
X-14662	21.975	18	0.233	21.975	17	0.186

**Table 3:** Results of horizontal pleiotropy by the MR-Egger intercept test and MR-PRESSO global test.

GDMs	MR-Egger intercept test			MR-PRESSO global test	
	Intercept	SE	P-value	Rss obs	P-value
Amino acid					
threonine	-0.061	0.098	0.545	6.05	1.000
N−acetylalanine	-0.170	0.442	0.705	23.31	0.546
indolepropionate	0.005	0.050	0.929	12.38	0.811
3−(3−hydroxyphenyl)propionate	-0.003	0.094	0.975	8.92	0.605
Cofactors and vitamins					
X−11593−−O−methylascorbate	0.008	0.026	0.766	42.43	0.832
Lipid					
dodecanedioate	-0.173	0.155	0.315	3.34	0.889
chiro−inositol	-0.135	0.071	0.085	17.03	0.324
15−methylpalmitate (isobar with 2−methylpalmitate)	0.041	0.062	0.519	12.87	0.624
Peptide					
pyroglutamylglycine	0.231	0.180	0.328	4.75	0.485
Xenobiotics					
saccharin	-0.075	0.082	0.384	3.65	0.972
glycerol 2−phosphate	-0.011	0.034	0.743	37.02	0.315
N−(2−furoyl)glycine	0.010	0.046	0.827	21.14	0.821
homostachydrine	0.294	0.151	0.124	6.69	0.507
salicyluric glucuronide	0.021	0.083	0.804	9.84	0.722
Unknown					
X−04495	-0.038	0.106	0.729	15.28	0.277
X−05907	0.002	0.075	0.983	12.37	0.591
X−09706	0.032	0.067	0.637	23.23	0.397
X−10510	0.006	0.061	0.920	15.70	0.838
X−02249	-0.119	0.102	0.267	12.86	0.563
X−11412	-0.033	0.037	0.376	40.29	0.774
X−11437	-0.039	0.062	0.538	21.45	0.213
X−11470	-0.019	0.094	0.844	22.48	0.161
X−11805	-0.005	0.060	0.933	4.44	0.949
X−12093	0.045	0.060	0.465	9.17	0.797
X−12206	0.230	0.119	0.084	14.04	0.357
X−12236	0.005	0.111	0.965	10.37	0.464
X−12524	0.026	0.056	0.649	31.70	0.510
X-12704	0.058	0.106	0.596	7.13	0.777
X-12776	0.131	0.158	0.559	NA	NA
X-12786	0.066	0.073	0.395	17.77	0.186
X-12844	0.011	0.096	0.913	16.11	0.540
X-13619	0.005	0.060	0.940	27.38	0.747
X-14662	0.001	0.050	0.987	23.81	0.306

### 3.4 Genetic variants for determining the causality of the association

Latent genetic variants were further delved into, which might cast a decisive role in identifying causal relationship between GDMs and POI. N-acetylalanine, a critical serum metabolite revealed by our study, was spotlighted. SNPs that exert an essential effect in the causal relationship between N-acetylalanine and POI were deeply investigated. Among the 23 SNPs constituting the IVs for N-acetylalanine, rs11144136 exhibited the most significant correlation signal with the largest association coefficient (β = 0.0097; standard error = 0.018; *P* = 4.82E-08, **Table 4**). Intriguingly, its strong effect on POI was also captured by our study (β = 0.3626; standard error = 0.1407; *P* = 0.099). Similarly, rs2019107 (β =-0.2666; standard error =0.0915; *P* =0.0036) and rs11606364 (β =0.4646; standard error =0.1951; *P* = 0.0173) were also significantly linked to POI. These SNPs may shed light on the underlying pathophysiological mechanisms of POI, as well as offer valuable insights into the identification of diagnostic and therapeutic targets.

**Table 4:** Genetic predictors of N−acetylalanine and their association with POI.

SNP	CHR	EA	RA	N−acetylalanine				POI			
				EAF	Beta	SE	*P* value	EAF	Beta	SE	*P* value
rs11144136	9	T	G	0.1375	0.0097	0.0018	**4.862E-08**	0.1111	0.3626	0.1407	**0.0099**
rs10125203	9	C	G	0.8698	0.009	0.0018	**4.109E-07**	0.8481	-0.0132	0.1248	0.9156
rs266742	3	T	G	0.7307	0.0082	0.0016	**5.676E-07**	0.7156	0.1562	0.0997	0.1174
rs1554074	12	A	G	0.1925	0.0086	0.0017	**6.392E-07**	0.1666	0.0389	0.1193	0.7442
rs6690829	1	T	C	0.24	-0.008	0.0016	**1.242E-06**	0.2599	-0.0233	0.1016	0.8183
rs11045425	12	A	G	0.1266	0.0086	0.0018	**1.832E-06**	0.1189	-0.0302	0.1394	0.8287
rs6462723	7	A	G	0.121	-0.0085	0.0018	**1.921E-06**	0.1023	-0.1744	0.1471	0.2360
rs9663814	10	A	G	0.6175	0.0076	0.0016	**1.952E-06**	0.6392	0.0999	0.093	0.2826
rs2596194	15	A	T	0.1255	-0.0083	0.0018	**2.586E-06**	0.0903	-0.0501	0.1562	0.7482
rs7178551	15	T	C	0.1545	0.0081	0.0017	**2.664E-06**	0.1213	0.0377	0.1365	0.7822
rs10988352	9	T	G	0.1244	0.0084	0.0018	**3.086E-06**	0.1473	-0.0422	0.127	0.7398
rs2486925	1	T	G	0.1462	-0.0082	0.0018	**3.467E-06**	0.1172	0.0856	0.1387	0.5372
rs10090064	8	T	C	0.8026	0.0077	0.0017	**5.931E-06**	0.7717	0.0624	0.1066	0.5583
rs513209	11	A	C	0.8824	-0.0082	0.0018	**6.602E-06**	0.829	-0.0899	0.1192	0.4506
rs2019107	19	T	C	0.6257	-0.0078	0.0017	**6.655E-06**	0.594	-0.2666	0.0915	**0.0036**
rs6019504	20	A	G	0.8586	0.008	0.0018	**6.689E-06**	0.8234	0.2158	0.1182	0.0679
rs2993308	13	A	G	0.338	0.0075	0.0017	**6.833E-06**	0.2931	0.0984	0.0982	0.3160
rs10850426	12	T	G	0.1199	0.0081	0.0018	**6.845E-06**	0.0712	0.0427	0.1761	0.8082
rs7250750	19	T	G	0.8583	0.0081	0.0018	**7.029E-06**	0.8116	-0.0674	0.1146	0.5564
rs732426	17	A	G	0.8218	0.0077	0.0017	**7.541E-06**	0.7889	-0.0378	0.1104	0.7319
rs10757735	9	A	C	0.132	-0.008	0.0018	**7.911E-06**	0.1226	-0.0569	0.136	0.6756
rs662760	6	A	C	0.8723	0.0079	0.0018	**8.042E-06**	0.896	0.0651	0.1443	0.6519
rs11606364	11	T	C	0.1149	0.0084	0.0019	**9.266E-06**	0.05562	0.4646	0.1951	**0.0173**

Values in bold represent statistically significant data with P < 0.05.

### 3.5 Replication and meta-analysis

To bolster the robustness of our findings, we expanded our MR analysis by incorporating additional GWAS datasets for the positive metabolites identified in above study. For the 33 candidate metabolites under investigation, GWAS data from alternative sources were successfully retrieved for 8 of them. A meta-analysis was subsequently performed on the MR results from all available data sources for each metabolite (**Supplementary Figures S1**–**S8**). As anticipated, multiple candidate metabolites exhibited similar trends in causal associations with POI across different GWAS data sources, akin to the patterns observed using data from Shin et al., although the causal effects were not significant, likely due to substantial differences in sample size. Notably, the meta-analysis result further validated the protective effect of salicyluric glucuronide on POI (**Supplementary Figure S1**), indicating that a higher genetic predisposition for salicyluric glucuronide (OR: 0.49; 95% CI: 0.32–0.77; *P* =0.002) might reduce susceptibility to POI.

### 3.6 Evaluation of genetic correlation and directionality

LDSC analysis was applied to examine the genetic correlation between POI and candidate metabolites. Most metabolites, except for X-13619 (rg=-1.163, P=0.035) and X-11593—O-methylascorbate (rg=0.703, P=0.038), showed no significant genetic correlation with POI, suggesting that MR estimates for these metabolites are unlikely to be confounded by shared genetic factors (**Supplementary Table S1**). However, due to limitations such as low heritability and small sample sizes, several metabolites were not amenable to this analysis. Moreover, MR Steiger directionality tests were performed for these metabolites and interpreted the direction from these GDMs to POI as robust, proving that the inferred causal directions between our exposures and outcome are “True” (**Table 5**). Results of the reverse MR analysis also demonstrated no definitive evidence of causality between POI phenotype and candidate GDMs within the IVW model (**Supplementary Table S2**), which was further corroborated by three additional MR approaches (**Supplementary Table S3**). Cochran’s Q test, MR-Egger, and MR-PRESSO analyses showed no substantial heterogeneity or horizontal pleiotropy in reverse MR study (**Supplementary Tables S4**, **S5**).

**Table 5:** Estimation of the Steiger direction from GDMs to POI.

GDMs	SNP_r2.exposure	SNP_r2.outcome	Direction	Steiger P-value
Amino acid				
threonine	0.0838	0.0001	TRUE	5.46E-97
N−acetylalanine	0.0694	0.0003	TRUE	1.78E-97
indolepropionate	0.0933	0.0001	TRUE	1.85E-117
3−(3−hydroxyphenyl)propionate	0.3135	0.0001	TRUE	1.43E-77
Cofactors and vitamins				
X−11593−−O−methylascorbate	0.2847	0.0004	TRUE	0
Lipid				
dodecanedioate	0.0860	0.0001	TRUE	7.28E-70
chiro−inositol	0.2601	0.0002	TRUE	3.05E-125
15−methylpalmitate (isobar with 2−methylpalmitate)	0.0729	0.0001	TRUE	4.60E-85
Peptide				
pyroglutamylglycine	0.0793	0.0001	TRUE	1.37E-21
Xenobiotics				
saccharin	0.1681	0.0001	TRUE	3.68E-78
glycerol 2−phosphate	0.1795	0.0003	TRUE	8.31E-199
N−(2−furoyl)glycine	1.2440	0.0002	TRUE	NA
homostachydrine	0.0811	0.0001	TRUE	6.74E-29
salicyluric glucuronide	0.5540	0.0001	TRUE	6.16E-115
Unknown				
X−04495	0.0536	0.0002	TRUE	2.57E-64
X−05907	0.0703	0.0001	TRUE	8.74E-86
X−09706	0.0858	0.0002	TRUE	5.48E-111
X−10510	0.1001	0.0002	TRUE	1.27E-140
X−02249	0.0462	0.0001	TRUE	1.88E-65
X−11412	0.1838	0.0004	TRUE	5.70E-251
X−11437	0.0980	0.0002	TRUE	5.80E-102
X−11470	0.0694	0.0002	TRUE	8.25E-89
X−11805	0.0920	0.0001	TRUE	2.17E-51
X−12093	0.3136	0.0001	TRUE	1.92E-173
X−12206	0.0870	0.0002	TRUE	5.02E-50
X−12236	0.1242	0.0001	TRUE	9.77E-50
X−12524	0.1322	0.0003	TRUE	3.83E-180
X-12704	0.1463	0.0001	TRUE	8.91E-53
X-12776	0.0289	0.0000	TRUE	1.57E-22
X-12786	0.0618	0.0002	TRUE	9.79E-66
X-12844	0.0763	0.0002	TRUE	2.88E-102
X-13619	0.1332	0.0003	TRUE	6.72E-183
X-14662	0.1555	0.0002	TRUE	3.21E-121

SNP, single nucleotide polymorphism; CHR, chromosome; EA, effect allele; RA,reference allele; EAF, effect allele frequency.

### 3.7 Results of genetic colocalization analysis

Colocalization analysis, serving as a crucial supplemental analysis to support the validity of IV assumptions in MR analyses, was performed for the 33 candidate metabolites. Causal associations between multiple metabolites and POI were revealed to be driven by shared genetic variants within gene loci (**Supplementary Table S6**). For instance, the MR effect of N-acetylalanine, one of the most significant compounds identified in this study, on POI was driven by the shared lead SNP rs11143900 located on chromosome 9p21.2-PIP5K1B (**Supplementary Figure S9**).

### 3.8 Metabolic pathway analysis

Based on multiple known metabolites with established causal connections to POI revealed in our study, we pinpointed two metabolic pathways potentially implicated in the mechanism underlying POI pathogenesis (**Supplementary Table S7**). The biosynthesis of valine, leucine, and isoleucine (*P* =0.0052), alongside the metabolism of glycine, serine, and threonine (*P* =0.0214), emerge as potential biological pathways involved in the development of POI. Further research focusing on these pathways is anticipated to elucidate the underlying mechanisms of POI more deeply.

## 4 Discussion

An unbiased detection of causal associations between GDMs and POI was implemented by employing two-sample MR analysis. Using genetic variants as proxies, causal associations of 33 serum metabolites and POI were uncovered via IVW approach. Association between genetically determined higher levels of X-11437 and increased risk of POI development was consistently validated across four MR analysis methods. Besides, our study revealed 26 metabolites exhibiting significant signals in most of the vital MR methodologies, and the known metabolite N-acetylalanine was disclosed as a crucial serum metabolite linked to an increased risk of POI. Moreover, potential genetic variants responsible for causality were further screened. By replication and meta-analyses with additional metabolite GWAS datasets, Steiger tests, and reverse analyses, the robustness of our findings were significantly bolstered. The assessment of genetic correlation between metabolites and POI through LDSC rendered the MR estimates more credible. Colocalization analysis provided strong evidence for causal connections between these metabolites and POI from the perspective of shared genetic variants. Two specific metabolic pathways involved in the pathogenesis of POI were also revealed. Our study underscored the complex genetic underpinnings of POI and suggested potential biological mechanisms for its pathology.

To the best of our knowledge, this is the first large-scale MR analysis incorporating metabolomics and genomics to unravel the physiopathological mechanisms of POI. GWAS data were utilized to investigate the causality between serum GDMs and POI at the genetic level. By focusing on certain metabolites, our study might yield useful recommendations for the prevention, treatment, and management of POI. This study has bridged a gap in the relevant field with research that offers novel insights into the role of interactions between genetic and metabolic factors in the pathogenesis of POI.

On the basis of a panel of 23 genetic scores with different degrees of specificity for N-acetylalanine, the genetic correlation between elevated levels of N-acetylalanine and increased risk of POI was demonstrated in this study. Previous metabolite profiling studies have indicated the presence of N-acetylalanine in human amniotic fluid, which is of great potential significance for the diagnosis of maternal or fetal disease (43). Nevertheless, the link between N-acetylalanine and other reproductive disorders, such as POI, has never received sufficient clinical attention. Given that this study has unveiled, for the first time, that N-acetylalanine was the most influential known metabolite in the causal correlation with POI, it is imperative to conduct future large-scale clinical data collection and in-depth correlative studies to further support the potential role of N-acetylalanine in the diagnosis or treatment of POI.

Our study also pinpointed a handful of other known metabolites that are possibly implicated in POI, including chiro-inositol, pyroglutamylglycine, saccharin, etc. Chiro-inositol is a carbocyclic sugar polyalcohol and comprises several isomers such as D-chiro-inositol (44). With high levels negatively affecting the quality of oocytes and blastocysts (45), D-chiro-inositol performs an instrumental role in the maintenance of normal ovarian function and state of health, making strict regulation of its abundance a necessity (46). Possible deleterious effects of D-chiro-inositol on ovarian tissue revealed by prior studies are in line with the causality between this GDM and POI unearthed in our study (47). Pyroglutamylglycine, a dipeptide composed of glycine and 5-oxo-L-proline, was reported to be a risk factor for the development of pancreatic cancer in a large prospective survey (48), whereas the association between pyroglutamylglycine and other diseases remained unclear. This study was the first to propose this causative link between pyroglutamylglycine and POI, laying foundation for subsequent insightful studies on this metabolite and POI pathogenesis. Saccharin and its salts are artificial nonnutritive sweeteners. Accumulated studies have documented that saccharin has detrimental impacts on reproductive indices in senescent mice (49), induces sister chromatid exchanges in hamster and human cells, and functions as a weak carcinogen to cause cytogenetic alterations (50). Sodium saccharin has also formerly been disclosed to exert an unfavorable biological influence on ovarian estrus in rats, with an increasing percentage of abnormal cycles and a growing number of ovarian cysts (51). As a further complement to the previous findings, our study shed the first light on the causal effect between saccharin and POI, supporting the harmful effects of saccharin on ovarian function.

In addition, it is worthy of attracting sufficient focus that this study has uncovered several interesting GDMs categorized as ‘unknown’, including X-11437 which exhibited robustly significant causal association with POI. Despite the fact that their chemical properties have not been clearly ascertained to date, causal associations between these metabolites and POI were revealed. We enrolled these “unknown” metabolites in our study as they still hold the attention of other researchers, expecting that these GDMs will gain wider attention in the future to yield valuable information.

Genetic variants associated with variation in the crucial target metabolite N-acetylalanine were also further drilled down. One of the N-acetylalanine IVs, SNP rs11045425, is situated within gene *SLCO1C1*. It has been reported that thyroid dysfunction is implicated in POI, and *SLCO1C1* can encode extremely pivotal transporter proteins of thyroid hormones, which are essential for ovarian function (52, 53). Apart from that, rs6690829, another IV for N-acetylalanine, lies on gene *KIF26B*, whose up-regulation correlates with cancer cell function in ovarian cancer (54). Although further evidence for the correlation of *KIF26B* with POI is lacking, associations discovered in this study might offer new clues for understanding the potential molecular mechanisms of POI. Similarly, rs10757735 is located on *LINGO2*, and studies have probed that *LINGO2* is a critical gene related to the development of embryos and oocytes (55, 56), indicating *LINGO2* might perform an influential role in the pathogenesis of POI. It is noteworthy that another instrumental variable for N-acetylalanine, rs732426, is located in *MYH13*. No link has been previously established between MYH13 and POI, but the Human Protein Atlas database (https://www.proteinatlas.org/) reports enrichment of *MYH13* in oocytes at the single-cell level, suggesting that *MYH13* may function in oocyte depletion in the ovary. These connections disclosed in this study serve to bring a completely novel perspective to the further excavation of pathogenic mechanisms for POI.

Moreover, the colocalization analysis revealed that N-acetylalanine shared a locus with POI, rs11143900, located on chromosome 9p21.2 within PIP5K1B. PIP5K1B has been reported to potentially function coordinately and/or redundantly in the maintenance of sperm number and morphology during spermatogenesis (57). The lack of research on PIP5K1B in ovarian-related fields warrants further attention in future studies.

However, this study has several limitations. First, our research identified metabolites causally associated with POI based on a robust MR approach, but the findings require to be further validated against experimental data. Second, the strength of IVs relies on the sample size of GWAS, therefore a larger amount of data should be gathered to improve the accuracy of generated GDMs. Third, GWAS analysis of serum metabolites utilized in this study was based on a European population, so it is inconclusive whether the results can be expanded to individuals of non-European ancestry owing to genetic differences between races.

## 5 Conclusion

By integrating comprehensive approaches including replication, meta-analysis, LDSC genetic correlation, colocalization, and metabolic pathway analysis, our MR study delivered an unconfounded estimation of the causality between multiple GDMs and POI. N-acetylalanine stands out as a key pathogenic metabolite linked to POI, with novel metabolites like X-11437 also showing strong associations. The exploration into genetic variations related to these GDMs enriched our understanding. This work not only underscored the significance of merging genomics and metabolomics to uncover disease mechanisms but also highlighted potential targets for POI treatment and prevention.

## Data availability statement

Publicly available datasets were analyzed in this study. This data can be found here: Data of POI can be publicly accessible on MRC Integrative Epidemiology Unit (https://gwas.mrcieu.ac.uk/). GWAS data for GDMs can be retrieved from Metabolomics GWAS server (https://metabolomics.helmholtz-muenchen.de/gwas/), and GWAS catalog database (https://www.ebi.ac.uk/gwas/home).

## Author contributions

SC: Data curation, Formal analysis, Investigation, Methodology, Software, Writing – original draft, Writing – review & editing. ZhZ: Data curation, Investigation, Resources, Writing – review & editing. ZiZ: Data curation, Formal analysis, Visualization, Writing – review & editing. YL: Visualization, Writing – review & editing. SS: Writing – original draft, Writing – review & editing. KH: Software, Writing – review & editing. QY: Formal analysis, Validation, Writing – review & editing. YG: Conceptualization, Formal analysis, Investigation, Methodology, Supervision, Visualization, Writing – review & editing.
