# Supplementary material for: Non-targeted metabolomics revealed novel links between serum metabolites and primary ovarian insufficiency: a Mendelian randomization study
Source: Front Endocrinol (Lausanne). 2024 Apr 26;15:1307944. doi: 10.3389/fendo.2024.1307944 (PMC11082646; doi:10.3389/fendo.2024.1307944)
Supplement: Supplementary file 1 [file DataSheet_1.docx]

**Supplementary Figures**


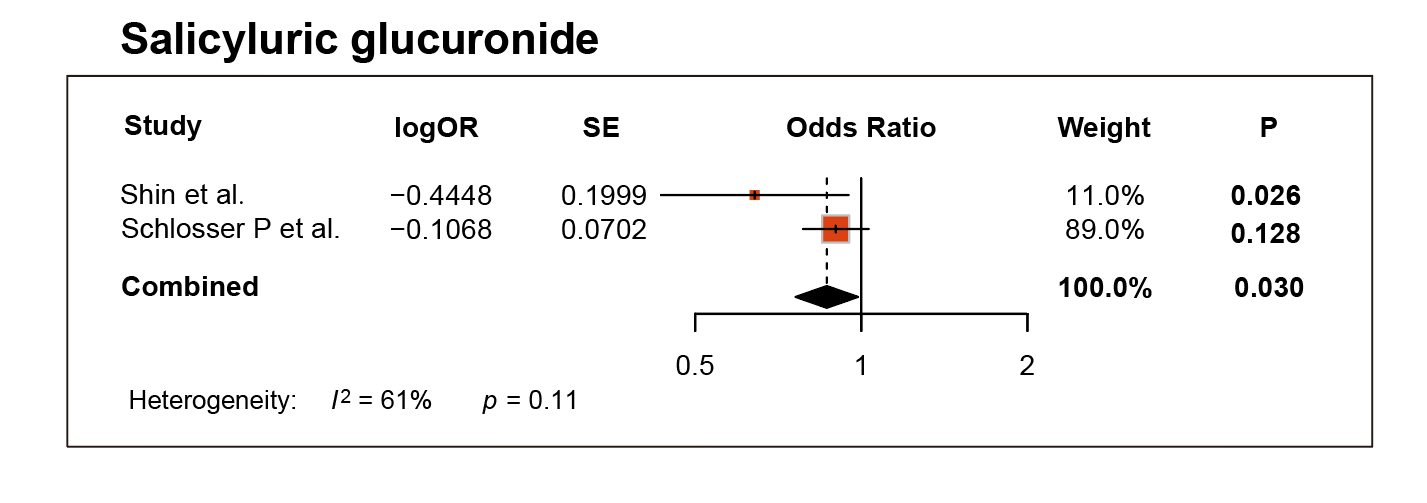


**Figure S1. Meta‐analysis of salicyluric glucuronide and POI.** The study denoted as "Shin et al." pertains to the GWAS data of 486 metabolites cited within our article. "Schlosser P et al." corresponds to research derived from GWAS data registered in the GWAS Catalog database (GCST90265935). 95% CI, 95% confidence interval; OR, odds ratio.


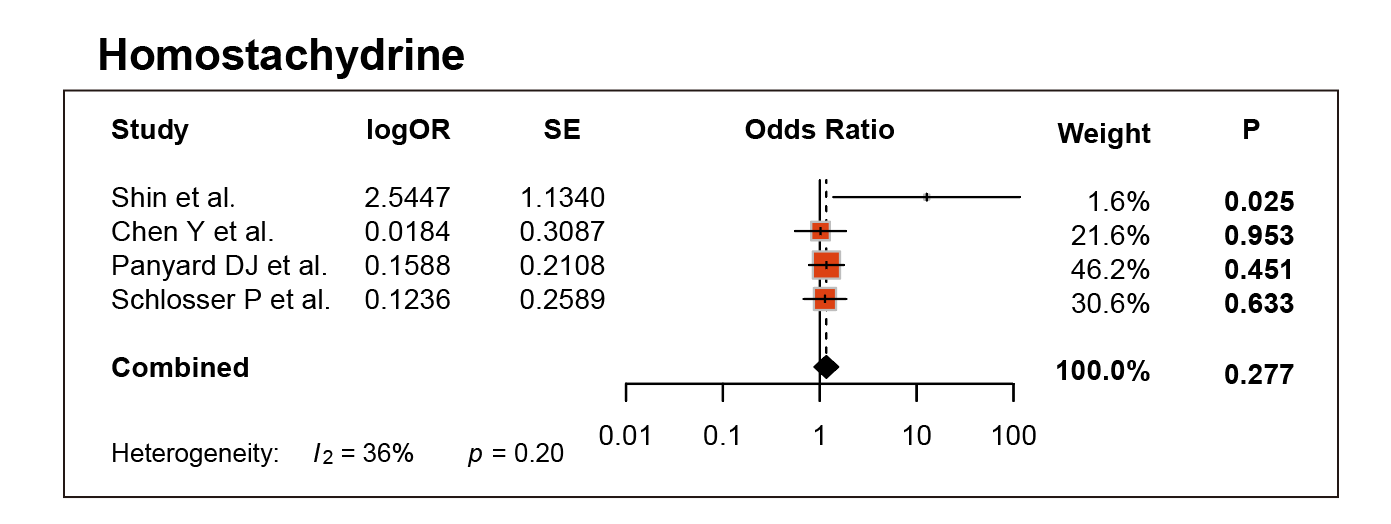


**Figure S2. Meta‐analysis of homostachydrine and POI.** The study denoted as "Shin et al." pertains to the GWAS data of 486 metabolites cited within our article. "Chen Y et al." corresponds to research derived from GWAS data registered in the GWAS Catalog database (GCST90199787). " Panyard DJ et al." corresponds to research derived from GWAS data registered in the GWAS Catalog database (GCST90026182). "Schlosser P et al." corresponds to research derived from GWAS data registered in the GWAS Catalog database (GCST90265352). 95% CI, 95% confidence interval; OR, odds ratio.


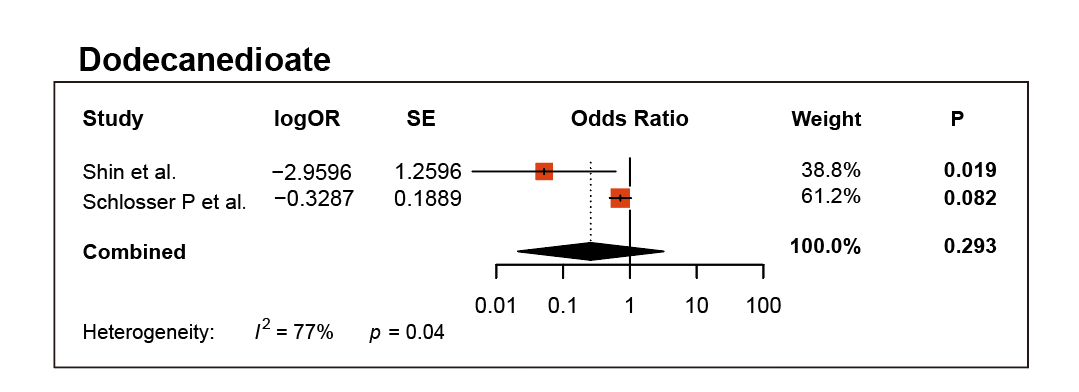


**Figure S3. Meta‐analysis of dodecanedioate and POI.** The study denoted as "Shin et al." pertains to the GWAS data of 486 metabolites cited within our article. "Schlosser P et al." corresponds to research derived from GWAS data registered in the GWAS Catalog database (GCST90265055). 95% CI, 95% confidence interval; OR, odds ratio.


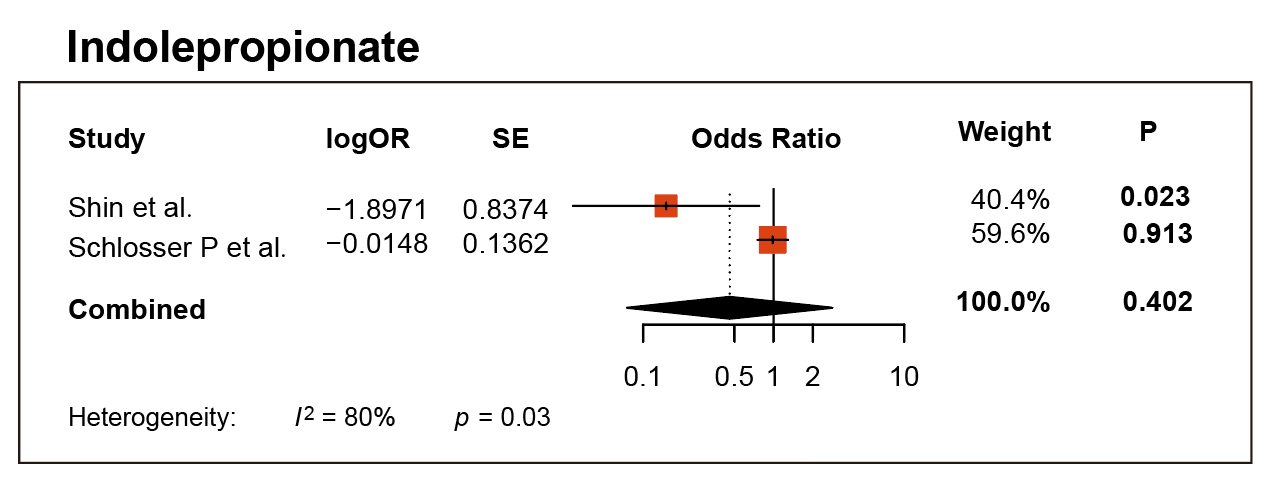


**Figure S4. Meta‐analysis of indolepropionate and POI.** The study denoted as "Shin et al." pertains to the GWAS data of 486 metabolites cited within our article. "Schlosser P et al." corresponds to research derived from GWAS data registered in the GWAS Catalog database (GCST90264496). 95% CI, 95% confidence interval; OR, odds ratio.


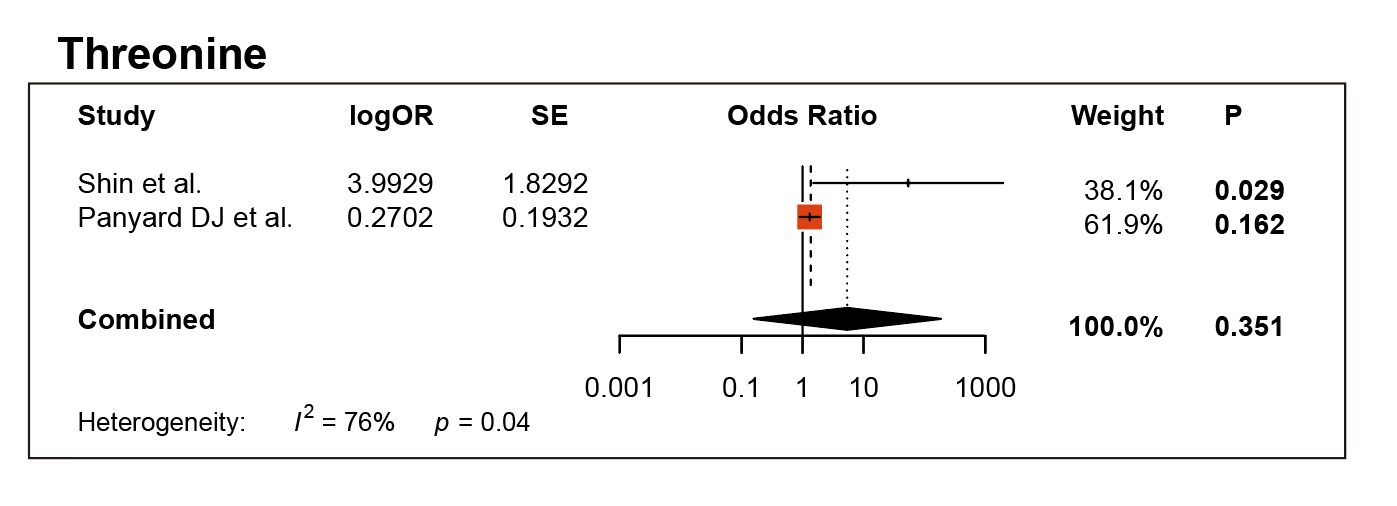


**Figure S5. Meta‐analysis of threonine and POI.** The study denoted as "Shin et al." pertains to the GWAS data of 486 metabolites cited within our article. "Panyard DJ et al." corresponds to research derived from GWAS data registered in the GWAS Catalog database (GCST90026276). 95% CI, 95% confidence interval; OR, odds ratio.


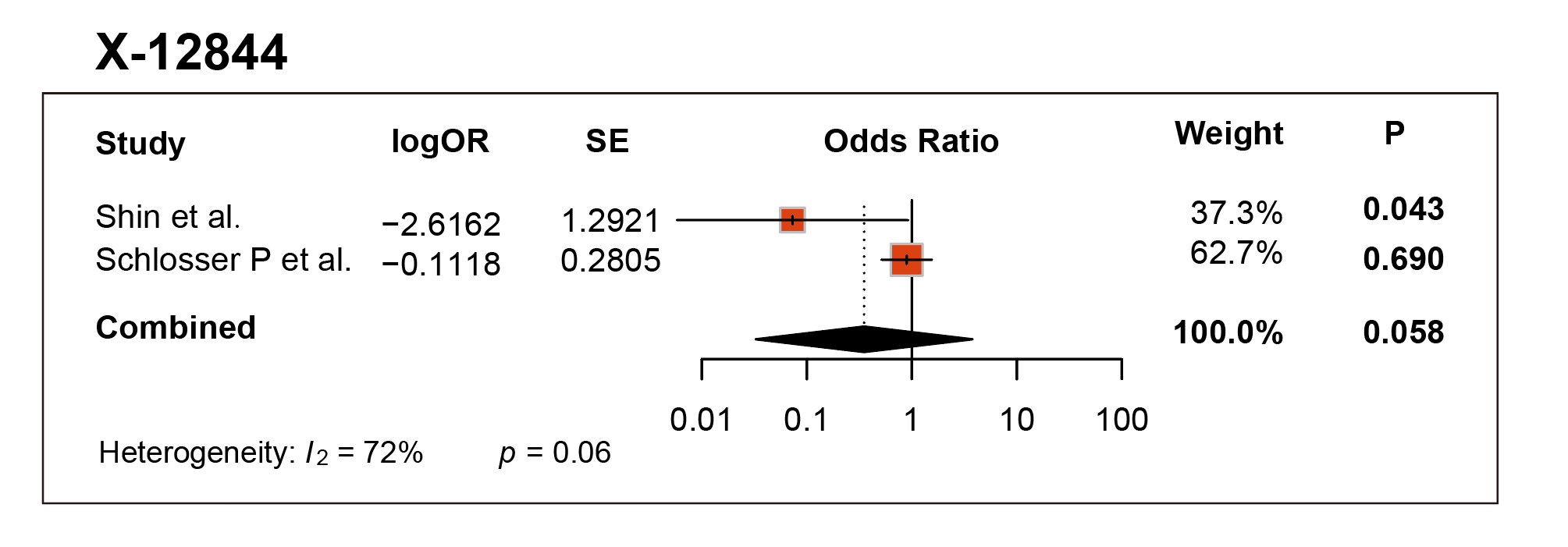


**Figure S6. Meta‐analysis of X-12844 and POI.** The study denoted as "Shin et al." pertains to the GWAS data of 486 metabolites cited within our article. "Schlosser P et al." corresponds to research derived from GWAS data registered in the GWAS Catalog database (GCST90266293). 95% CI, 95% confidence interval; OR, odds ratio.


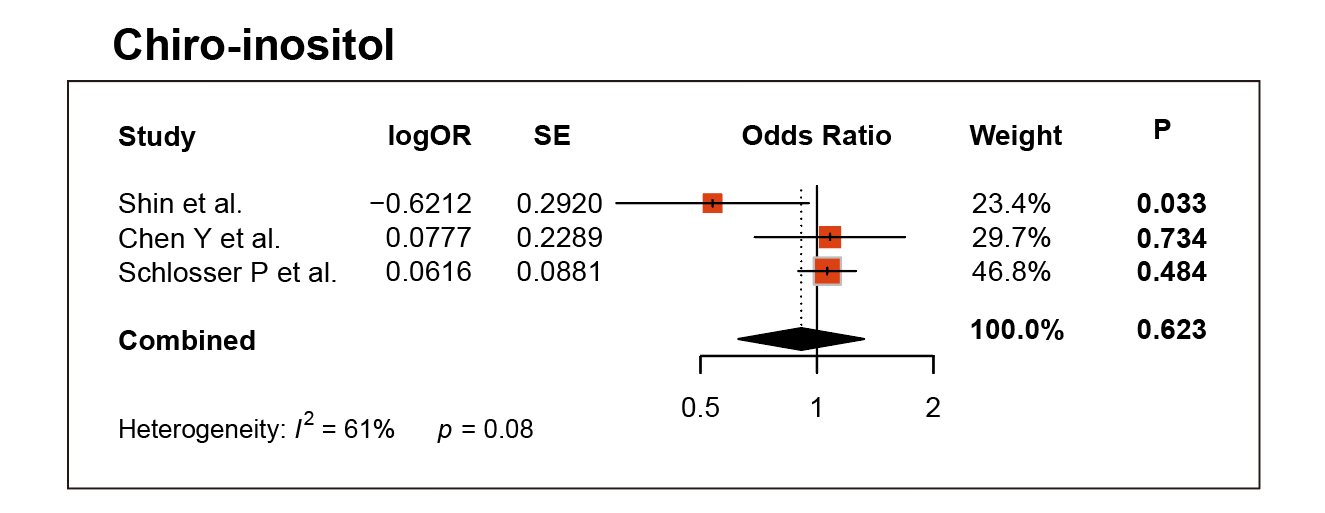


**Figure S7. Meta‐analysis of chiro-inositol and POI.** The study denoted as "Shin et al." pertains to the GWAS data of 486 metabolites cited within our article. "Chen Y et al." corresponds to research derived from GWAS data registered in the GWAS Catalog database (GCST90199843). "Schlosser P et al." corresponds to research derived from GWAS data registered in the GWAS Catalog database (GCST90264914). 95% CI, 95% confidence interval; OR, odds ratio.


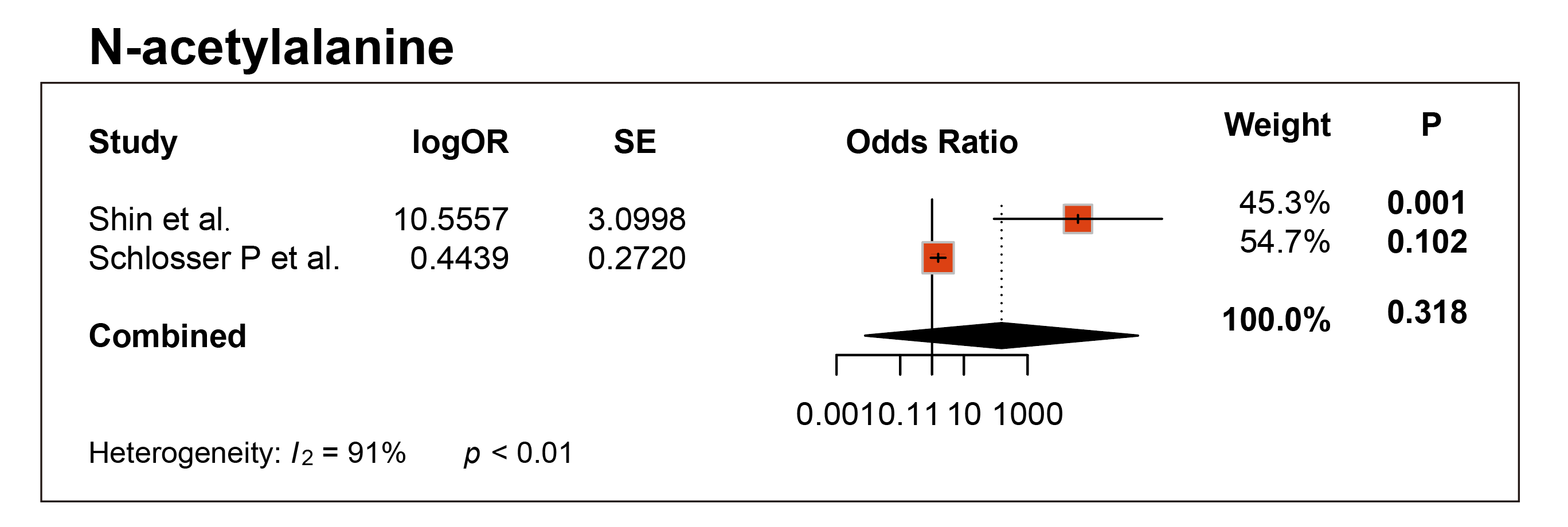


**Figure S8. Meta‐analysis of N-acetylalanine and POI.** The study denoted as "Shin et al." pertains to the GWAS data of 486 metabolites cited within our article. "Schlosser P et al." corresponds to research derived from GWAS data registered in the GWAS Catalog database (GCST90265528). 95% CI, 95% confidence interval; OR, odds ratio.


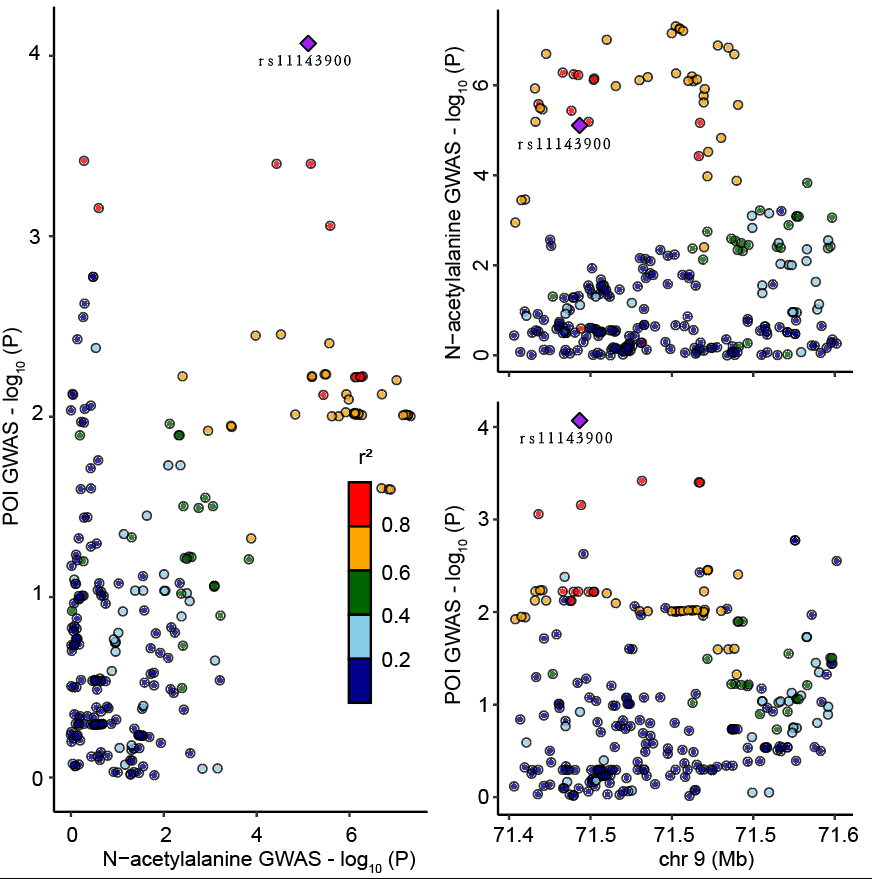


**Figure S9. Regional Manhattan Plots Illustrating genetic evidence of colocalization between total body N-acetylalanine and POI.** Variants are colored by linkage disequilibrium with rs11143900.
